# Solution and Suspension

**Published:** 1881-10

**Authors:** 


### SOLUTION AND SUSPENSION.

Some apparently dilettante but really very valuable work may be accomplished by amateur microscopists were they to examine carefully the appearances of every tincture, fluid extract or liquid mixture on their shelves, and report the result. Just as it is impossible to draw the line anywhere in nature between existing conditions, so solutions merge into mixtures, and many really suspended preparations in liquid pass for solutions simply because the minute division of particles admits of no optical clearness until magnified. Tyndall illustrated this peculiarty of mixtures by means of a helix of wires surrounding a vial of ferric sulphate in solution. As soon as a current of electricity passed over the wire the opaque liquid cleared up and light passed freely through the vial, owing to the axial setting of the particles in the path of the light rays. No such work as that suggested has been attempted, and were such appearances detailed in our dispensatories and pharmacopæias, a valuable addition would be made to our testing resources. Of course, the powers used should be noted with each description. In very many cases negative results would be obtained, but a multitude of so-called solutions would be placed among mixtures after such work.

Dentists are content to call the mixture of metals which they use as plugs for carious teeth, an amalgam, after expressing the murcury from the mixture with their pliers. Now, were they to note the appearance of this amalgam under a low power and then under higher, they would become skeptical concerning the definiteness of the combination, and some of the bad results from use of this murcurial plug would be accounted for. Similarly the irritating and other undesirable properties of some galenical preparations would be explained by differences in division of this suspended drug.—*The Druggist*, September, 1881.

